# SMN deficiency causes pain hypersensitivity in a mild SMA mouse model through enhancing excitability of nociceptive dorsal root ganglion neurons

**DOI:** 10.1038/s41598-019-43053-5

**Published:** 2019-04-24

**Authors:** Ruobing Qu, Fuping Yao, Xiaomin Zhang, Yuan Gao, Tong Liu, Yimin Hua

**Affiliations:** 1 Jiangsu Key Laboratory of Neuropsychiatric Diseases, Department of Neurology and Suzhou Clinical Research Center of Neurological Disease, the Second Affiliated Hospital of Soochow University, 1055 Sanxiang Road, Suzhou, 215004 China; 2 Institute of Neuroscience, Soochow University, 199 Renai Road, Suzhou, Jiangsu 215123 China

**Keywords:** Chronic pain, Molecular neuroscience, Sensory processing

## Abstract

Spinal muscular atrophy (SMA) is a devastating motor neuron degeneration disease caused by a deficiency of the SMN protein. Majority of patients also suffer from chronic pain. However, the pathogenesis of pain in the context of SMA has never been explored. In this study, using various pain tests, we found that a mild SMA mouse model presents with multiple forms of pain hypersensitivity. Patch-clamp recording showed that nociceptive neurons in SMA mouse dorsal root ganglia (DRGs) are hyperexcitable and their sodium current densities are markedly increased. Using quantitative RT-PCR, western blotting and immunofluorescence, we observed enhanced expression of two main voltage-gated sodium channels Na_v_1.7 and Na_v_1.8 in SMA mouse DRGs, which is at least in part due to increase in both expression and phosphorylation of NF-κB p50/p65 heterodimer. Moreover, we revealed that plasma norepinephrine levels are elevated in SMA mice, which contributes to mechanical hypersensitivity via the β2-adrenergic receptor. Finally, we uncovered that β2-adrenergic signaling positively modulates expression as well as phosphorylation of p50 and p65 in SMA mouse DRGs. Therefore, our data demonstrate that SMA mice, similar to humans, also develop pain hypersensitivity, and highlight a peripheral signaling cascade that elicits the mechanical sensitization in the mouse model, suggesting potential targets for therapeutic intervention.

## Introduction

Spinal muscular atrophy (SMA) is an autosomal recessive motor neuron disorder. It is the most common genetic killer of infants, caused by mutations in the *survival of motor neuron 1* (*SMN1*) gene^1^. SMA is characterized by degeneration of α-motor neurons in the spinal cord, which results in progressive muscle weakness and paralysis^2^. Humans have a unique paralogous *SMN2* gene; both genes encode an identical protein SMN. However, owing to two key nucleotide changes, C6T in exon 7 and G-44A in intron 6, *SMN2* is alternatively spliced with ~90% of transcripts being exon 7-skipped, giving rise to a truncated dysfunctional protein, SMNΔ7^3,4^. The limited full-length SMN protein expressed by *SMN2* is not sufficient to compensate for the deficit of *SMN1*. SMA is classically subdivided into four clinical types based on the severity and age of onset: Type I is most severe, type II is the intermediate form, type III is the mild form and type IV is the adult-onset form^5^.

As a ubiquitously expressed protein, SMN is essential for survival of all cell types. Inactivation of the mouse *Smn* (also called *Smn1*) gene, the only SMN-expressing gene in mice, is embryonically lethal^6^. SMN is a multifunctional protein, involved in various cellular activities, particularly RNA metabolic processes, including assembly of ribonucleoproteins (RNPs), such as the spliceosomal U small nuclear RNPs (snRNPs) and histone RNA processing U7 snRNP^7,8^, and transport of mRNAs into axonal growth cones for local protein translation^9^. To date, the molecular mechanisms underlying the selective degeneration of lower α-motor neurons remain elusive. However, numerous studies have delineated defects in multiple cell types, tissues and organs aside from spinal cord motor neurons in SMA patients and mouse models, particularly of severe forms^10–12^. Recently, several groups provided strong evidence that cell-nonautonomous mechanisms account for or contribute to spinal cord motor neuron death and/or motor dysfunction in mouse and *Drosophila* models^13–18^.

Widespread defects in sensory neurons have been observed in SMA patients and mouse models. For example, Anagnostou *et al*. observed that the conduction velocity of sensory and mixed nerves was reduced in type I patients^19^. Rudnik-Schoneborn *et al*. observed a much smaller nerve fiber density in the sural nerve in patients with pre-natal disease onset^20^. In severe mouse models, despite no loss of sensory neurons, their neurite outgrowth is reduced and sensory-motor connectivity is functionally impaired^21–23^. Surprisingly, Fletcher *et al*. recently revealed in SMA mice that dysfunction of proprioceptive neurons due to SMN deficit is the primary cause of motor neuron dysfunction and restoration of SMN in proprioceptive neurons ameliorates neuromuscular-junction function and motor behavior^14^. As humans and mice employ similar neurotransmitters in sensory-motor circuits, this finding demonstrates a critical role of sensory neurons in SMA pathogenesis.

Chronic pain is an unpleasant sensation that persists over a long period of time. It is a major comorbid condition in many diseases including neurological disorders^24^. Lager and Kroksmark reported that 71% of the adolescents with SMA and 41% of the adolescents with dystrophinopathy suffered chronic pain^25^. Among them, 59% reported weekly, daily or constant pain with episode durations ranging mostly from 1 to 12 hrs, and the most common pain sites were the neck, back and legs. Sitting, too much movement and being lifted and transferred have been recognized as the main factors to exacerbate pain^25^. Although the average pain intensities were graded as mild or moderate, it affected their general activities and mood^25^. Chronic pain can be caused by various factors, such as neuropathic or inflammatory in its origin. Understanding of the specific pathophysiological mechanisms of pain associated with a disease should shed light on design of therapeutic approaches for pain relief.

Pain in SMA mouse models has never been investigated. Based on previous studies, we hypothesize that nociceptive neurons in dorsal root ganglia (DRGs) might be functionally impaired in mouse models. The objectives of this study were to address if pain hypersensitivity occurs in SMA mice, and if so, to explore the underlying molecular mechanisms. Using five pain tests, we revealed that multiple forms of pain sensitivity are markedly increased in a mild SMA mouse model. Using patch-clamp recordings we found hyperexcitability of nociceptive DRG neurons and increase in sodium current densities. Furthermore, we identified a signaling cascade from elevated plasma norepinephrine (NE) to enhanced NF-κB activity that upregulates sodium channels and thus enhances excitability of DRG neurons in SMA mice. Our data may have clinical value in search for treatment to alleviate chronic pain in SMA patients.

## Results

### Pain hypersensitivity occurs in SMA mice

**Figure 1:**
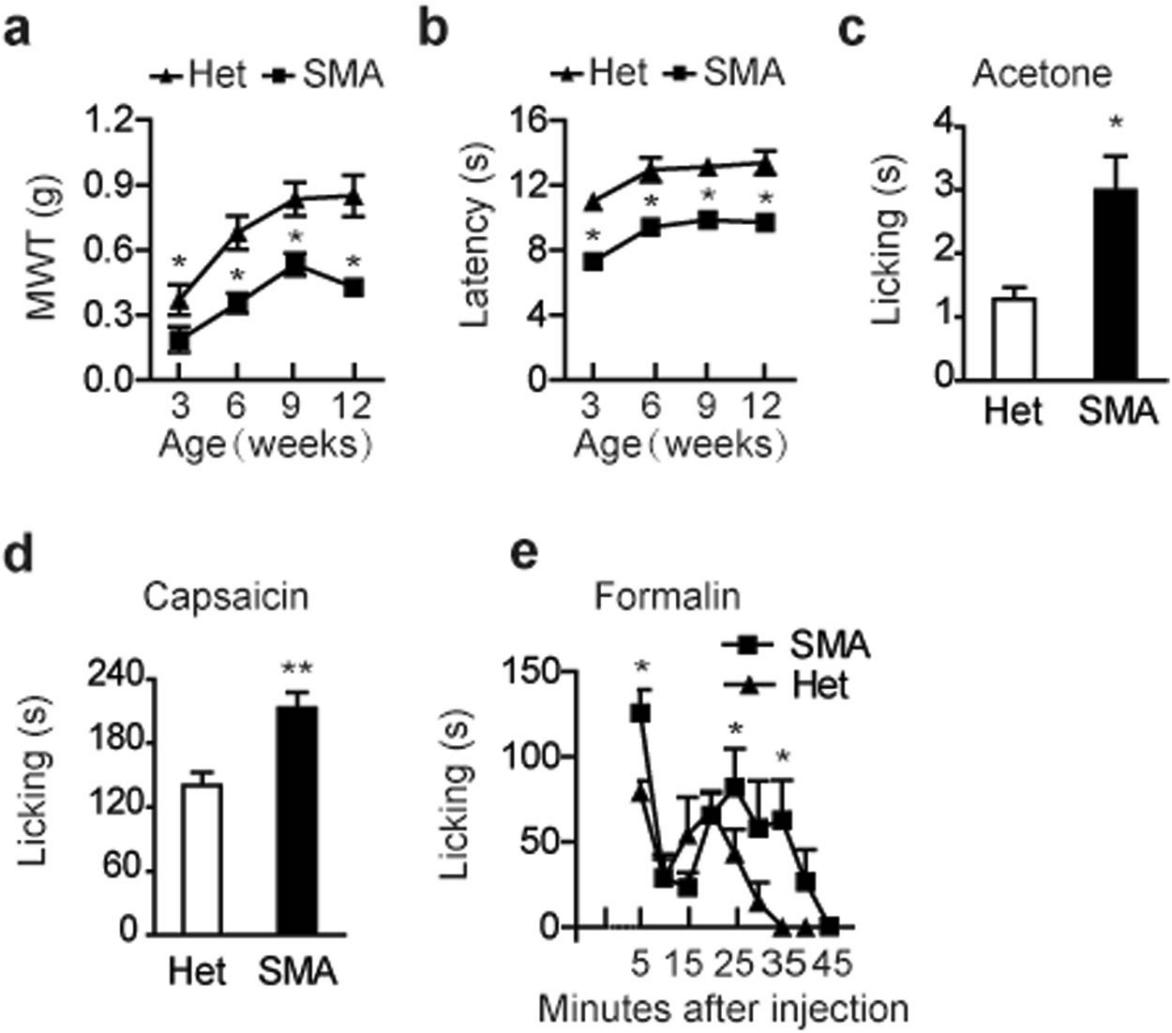
Detection of pain hypersensitivity in SMA mice. (**a**) Von Frey filament test (0.07–1.4 g) was used to evaluate mechanical sensitivity of SMA and control heterozygous (Het) mice with four age groups: 3-, 6-, 9- and 12-week old, respectively (n = 12 per group). At all ages, SMA mice displayed pronounced decrease in mechanical withdraw threshold (MWT). (**b**) Hargreaves test was used to evaluate heat sensitivity and mice were grouped (n = 6 per group) the same as in von Frey test. Similarly, all SMA mice displayed pronounced decrease in thermal withdraw latency (s). (**c**–**e**) 9-week old mice were either treated with 25 μl acetone (n = 7 per group; SMA versus control) on the plantar surface of hind paws, or intraplantarly injected with 10 µl capsaicin solution at 0.2 µg/µl (n = 5 per group) or with 20 µl of 5% formalin solution (n = 6 per group). The time spent in nocifensive behaviors (licking, etc.) was recorded. For all panels, *P < 0.05, **P < 0.01, compared to Het controls.

A high percentage of patients with SMA suffer from chronic pain^25^. It prompted us to ponder whether such a phenomenon is also present in SMA mouse models. To this purpose, we chose a mild Taiwanese mouse model (*Smn*^−/−^, *SMN2*^2TG/2TG^) to test sensitivity of pain^26,27^. The reason that we did not choose severe models is because they die prematurely and are technically difficult to analyze the pain profiles. The mild model appears to live and breed normally but develops distal necrosis on the tail and ear pinnae in the 4th week after birth, leading to gradual loss of the two tissues. To evaluate mechanical sensitivity, we employed a commonly used von Frey test, in which a series of filaments with different forces were applied to mouse hind paws so that the mechanical withdraw threshold of hind limbs could be measured. Four age groups (3-, 6-, 9-, and 12-week old, respectively) with twelve mice per group were tested and asymptomatic heterozygous mice (*Smn*^+/−^, *SMN2*^2TG/2TG^) of the same ages were used as controls. Reponses to both innocuous (below-threshold) and noxious (above-threshold) mechanical stimuli in all groups of SMA mice were markedly increased than those in control mice (Fig. 1a; Supplemental Fig. S1), suggesting that SMA mice bear both mechanical allodynia and hyperalgesia. We next determined heat sensitivity using Hargreaves test, in which radiant heat was applied to the plantar surface of mouse hind paws and paw withdraw latency was measured. Four age groups of mice, the same as above, were tested. Similar to mechanical sensitivity, heat sensitivity, reflected by heat withdraw latency, was also markedly increased in all SMA mice (Fig. 1b**)**. Heat sensitivity was also assessed with the conventional hot plate test^28^, which further indicates that thermal allodynia and hyperalgesia occur in SMA mice (Supplemental Fig. S2). Of note, all the mice in the 3-week age group still had an intact tail and ear pinnae, and thus mechanical and heat hypersensitivity observed in this group indicates that distal necrosis is not a cause of pain hypersensitivity.

We further compared cold sensitivity between 9-week old SMA and heterozygous mice using acetone test, in which 25 μl acetone was applied to the plantar surface of hind paws, and time spending in flinching, licking or biting of their hind paws was recorded. The duration of pain-like behaviors of SMA mice increased by >2 fold (Fig. 1c). The nocifensive response induced by capsaicin in mice at 9 weeks old was also assessed. After intraplantar injection of 10 µl capsaicin solution (0.2 µg/µl), time spent in flinching, licking or biting of the injected paw by SMA mice was much longer than that by heterozygous mice (Fig. 1d). Furthermore, we performed formalin test on 9-week old mice. After intraplantar injection of 20 µl of 5% formalin, time course of nocifensive behaviors was analyzed (Fig. 1e). Pain sensitivity in formalin-treated SMA mice was markedly increased in the first phase (0–5 min post the injection) as well as on some time points in the second phase (10–40 min post the injection).

### DRG nociceptive neurons are hyperexcitable

**Figure 2:**
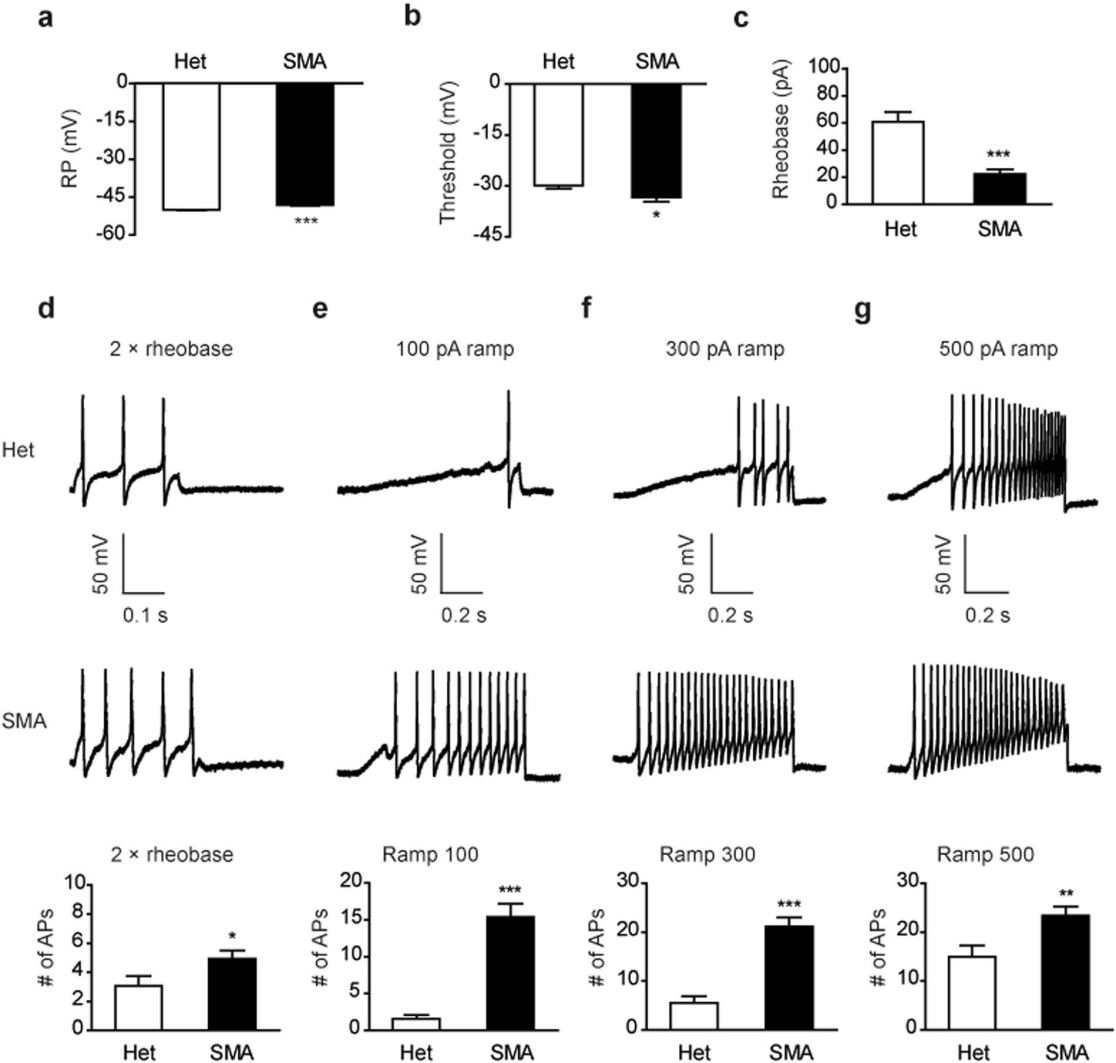
Analysis of excitability of SMA mouse DRG neurons. Small-diameter L4–L6 DRG neurons were isolated from 8 SMA mice and 9 control heterozygous (Het) mice at the age of 9 weeks. The RP (**a**), AP threshold (**b**) and rheobase (**c**) were analyzed. SMA mouse neurons had a significantly higher RP (48.0 mV, n = 13) than controls (50.0 mV, n = 14). The AP threshold and rheobase were significantly lower in SMA mice (−33.4 mV and 22.3 pA, respectively) than those in heterozygous controls (−29.8 mV and 60.7 pA, respectively). (**d**) The number of APs for the DRG neurons evoked by 2× rheobase current stimulation in 510 ms was significantly increased in SMA mice (5) compared to heterozygous mice (3). (**e**–**g**) The number of Aps for the neurons evoked by 100, 300 or 500 pA current ramp (1300 ms) was robustly increased in SMA mice compared to controls. Quantitation of APs in panels (e–g) is shown in histograms below. For all panels, n = 13–14 neurons per group, *P < 0.05, **P < 0.01, and ***P < 0.001 versus controls.

The above pain tests show the presence of various forms of allodynia and hyperalgesia in the SMA model, suggesting that pain-associated neurons may be hyperexcitable. To test this hypothesis, we dissected out DRGs of lumbar segments L4–L6 that receive stimuli from mouse hind limbs. Mice were 9 weeks old. Small-diameter single neurons (20–25 μm), representing afferent neurons responsible for pain sensation, i.e. nociceptive neurons^29,30^, were isolated for excitability analysis using whole-cell patch clamp recording. Under current-clamp, we observed a small but significant hyperpolarization of rest potential (RP) in SMA mouse nociceptive neurons when currents were held at 0 pA (−50.0 mV in controls versus −48.0 mV in SMA mice, Fig. 2a). All recorded neurons fired action potentials (APs) when injected with currents from −60 pA to +100 pA with 10 pA step for 510 ms. The AP threshold was −33.4 mV for SMA mouse neurons, significantly lower than −29.8 mV for control neurons (Fig. 2b). The minimal current required for triggering APs, rheobase, was 22.3 pA for SMA mouse neurons, which was a 2.7-fold reduction compared to 60.7 pA for control neurons (Fig. 2c). In addition, the number of APs in response to 2× rheobase current stimulation in SMA and age-matched control mice was 3 and 5, respectively, representing a dramatic increase (Fig. 2d). Finally, we examined excitability of these neurons with 1300 ms current ramp stimulations. The number of APs evoked by 100, 300, or 500 pA current ramp was increased from 1.5, 5.5, or 14.9 in control neurons to 15.3, 21.1, or 23.3 in SMA neurons, respectively. Therefore, the number of APs was robustly increased in SMA mouse DRG neurons in all tested conditions. Taken together, these electrophysiology data indicate that the DRG neurons associated with pain are hyperexcitable in SMA mice.

### Na^+^ current densities of DRG neurons are increased in SMA mice

**Figure 3:**
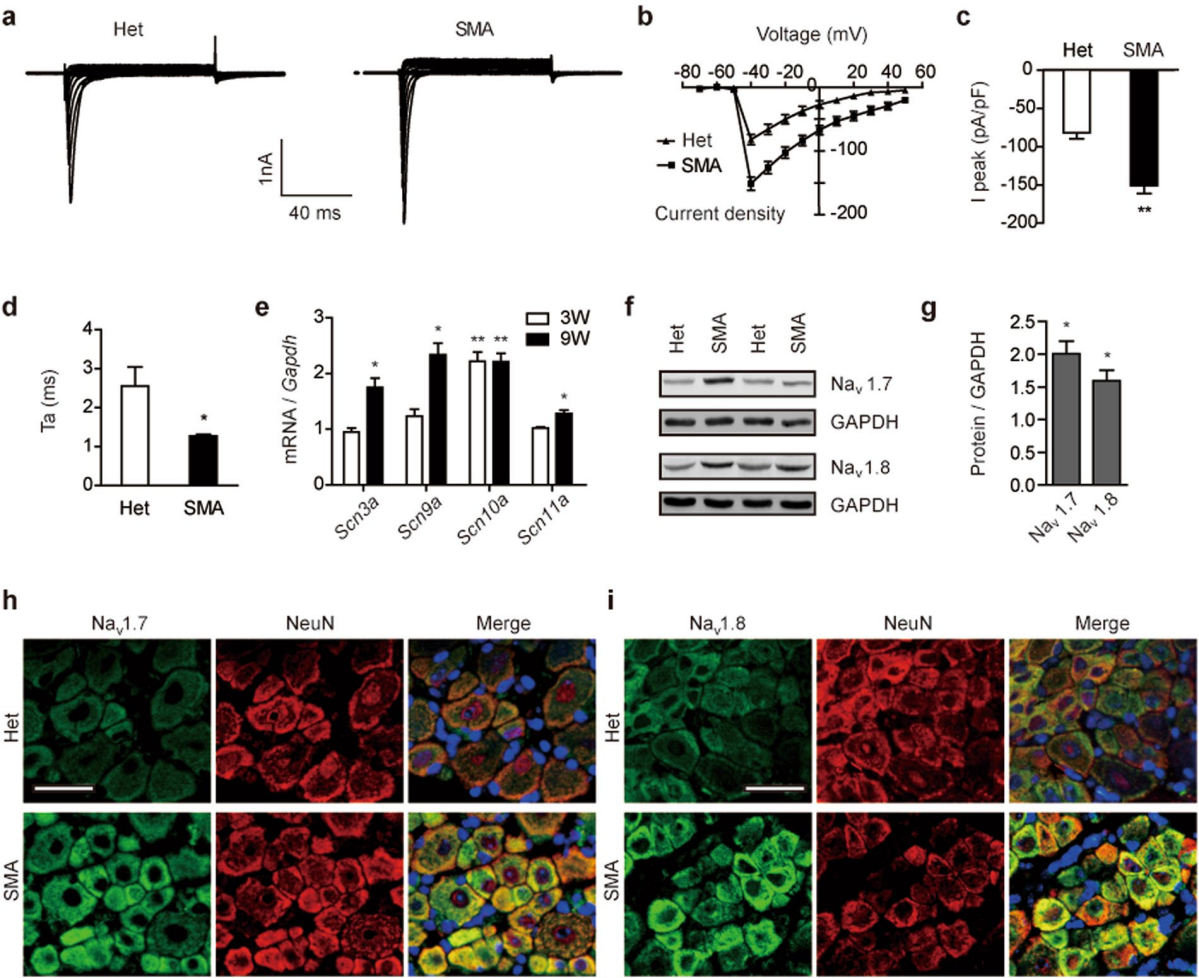
Na^+^ currents and expression of related genes in SMA mouse DRG neurons. (**a**) Small-diameter L4–L6 DRG neurons were isolated from 6 SMA mice and 7 heterozygous (Het) mice at the age of 9 weeks, and Na^+^ current densities of 11 neurons in each group were recorded. The membrane potential was held at −60 mV; voltage stimulation was from −70 to +50 mV with −10 mV increments and duration of 80 ms. (**b)** Current-voltage curves for Na^+^ currents as shown in panel (a) were plotted. Current was normalized to membrane capacitance. (**c)** Histogram of the peak amplitude of Na^+^ currents from panel (b). (**d**) Histogram shows the mean activation time (Ta) of Na^+^ currents between 10% and 90% of the peak value from panel (a). (**e**) Total 18 DRGs obtained from three mice were pooled together for each RNA or protein sample. *Scn3a*, *Scn9a*, *Scn10a* and *Scn11a* mRNA levels were analyzed with qRT-PCR using specific primers, normalized to *Gapdh* and presented as fold changes to Het (n = 4 per group). (**f**) Western blotting analysis of Na_v_1.7 (n = 3) and Na_v_1.8 (n = 4) protein levels in DRGs using specific antibodies. GAPDH was used as a loading control. (**g)** Quantitation of protein signals in panel (f) and unshown data; data were presented as fold changes to Het controls. (**h**–**i**) DRG sections, obtained from 5 SMA and 5 Het mice, were stained with anti-Na_v_1.7 or anti-Na_v_1.8 antibody (green). NeuN (red) was used as a neuronal marker. DAPI (blue) was used for nuclear counterstaining. Scale bar, 25 μm. Representative images are shown. For panels (c,d,e,g), *P < 0.05, **P < 0.01 versus Het.

Voltage-gated Na^+^ channels (VGSCs) are the main class of ion channels responsible for the generation and propagation of APs in neurons and implicated in chronic pain^31^. Therefore, we measured current densities of VGSCs in L4–L6 DRG neurons obtained from SMA mice (Fig. 3). Na^+^ currents were evoked by 13 test potentials from −70 to +50 mV in 10-mV increments for 80 ms, and then normalized to membrane capacitance. The peak amplitude of Na^+^ currents in neurons was drastically increased in SMA mice (−151.2 pA/pF) compared to that in control mice (−81.9 pA/pF) (Fig. 3a–c). We also analyzed the sodium current kinetics of VGSCs. As shown in Fig. 3d, the activation time of VGSCs between 10% and 90% of the peak value was 1.26 ms in SMA cells, significantly lower than 2.55 ms as observed in controls. These results indicate that hyperexcitability of DRG nociceptive neurons in SMA mice is mediated, at least in part, by sensitization of Na^+^ channel currents.

### Expression of Na_v_1.7 and Na_v_1.8 is increased in SMA mouse DRGs

As multiple VGSCs play critical roles in peripheral pain signal transduction, we speculate that upregulation of certain sodium channels may be the cause of elevated Na^+^ current densities in the DRG neurons. Four α-subunits of sodium channels Na_v_1.3, Na_v_1.7, Na_v_1.8 and Na_v_1.9 are highly expressed in DRGs and play crucial roles in nociception^31–34^; particularly, knockdown of tetrodotoxin (TTX)-sensitive Na_v_1.7 or TTX-resistant Na_v_1.8 displayed anti-allodynic and anti-hyperalgesic effects in rat pain models^35,36^. Therefore, we examined their expression changes in SMA mouse DRGs. Using quantitative RT-PCR (qRT-PCR), we observed a marked increase in expression of all four genes, particularly *Scn9a* that encodes Na_v_1.7 and *Scn10a* that encodes Na_v_1.8 in DRGs derived from 9-week-old SMA mice compared to heterozygote controls. *Scn9a* mRNA levels were increased by 2.3-fold and *Scn10a* by 2.2-fold, respectively (Fig. 3e). At the age of 3 weeks old, a similar 2.2-fold increase in *Scn10a* expression was observed; however, no appreciable increase in mRNA expression of *Scn3a*, *Scn9a* and *Scn11a* was detected at this age, suggesting *Scn10a* may be more sensitive to reduction of SMN levels. As *Scn9a* and *Scn10a* mRNA levels were robustly altered in DRGs of 9-week SMA mice, we further performed western blotting to examine their protein expression. Using specific polyclonal antibodies, we indeed observed a corresponding increase in Na_v_1.7 and Na_v_1.8 levels in SMA mouse DRGs (Fig. 3f,g). We also performed immunofluorescence on DRGs; NeuN was used as a neuronal marker. Neurons obtained from SMA mice had much stronger Na_v_1.7 and Na_v_1.8 signals than those obtained from control mice (Fig. 3h,i). Taken together, our data demonstrate that the increase in Na^+^ current densities is attributable to enhanced expression of sodium channels, particularly Na_v_1.7 and Na_v_1.8.

### NF-κB signaling is significantly increased in DRGs of SMA mice

**Figure 4:**
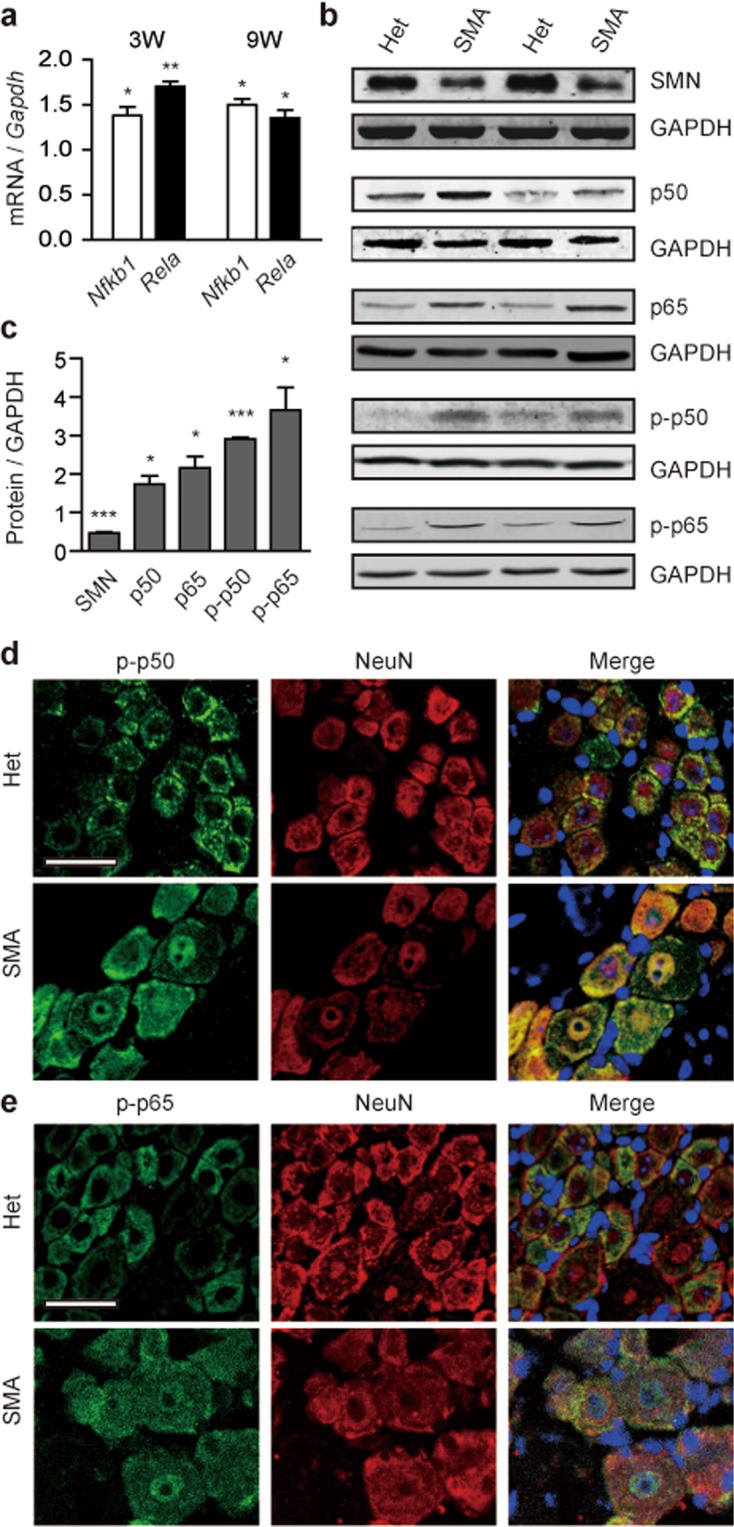
Both expression and phosphorylation of NF-κB subunits in SMA mouse DRGs were increased. L4–L6 DRGs were isolated from SMA and heterozygous (Het) mice at 9 weeks of age. Total 18 DRGs were pooled together for each RNA or protein sample. (**a**) *Nfkb1* and *Rela* mRNA levels were analyzed with qRT-PCR using specific primers and normalized to *Gapdh* (n = 4 per group). (**b**) Western blotting was used to analyze protein levels of SMN (n = 3), p50 (n = 4), p65 (n = 4), p-p50 (n = 3), and p-p65 (n = 4), respectively, in DRGs with specific antibodies. GAPDH was used as a loading control. **c** Fold changes of protein signals in SMA mice shown in panel (b). For panels (a,c), *P < 0.05, **P < 0.01, ***P < 0.001 versus Het. (**d**–**e)** DRG sections, derived from five SMA and five Het mice, were stained with anti-p-p50 antibody or anti-p-p65 (green) with NeuN (red) being a neuronal marker. DAPI (blue) was used to stain nuclei. Scale bar, 25 μm. Representative images are shown.

NF-κB regulates expression of an enormous number of genes associated with immunity, apoptosis, stress responses and differentiation^37,38^. Activation of NF-κB is also a cause of pain hypersensitivity in multiple pain models and diseases that are known for severe pain^39,40^. Two studies reported that the TNFα/NF-κB pathway stimulates expression of Na_v_1.7 and Na_v_1.8 in DRG neurons in rat neuropathic pain models^41,42^. Interestingly, we have recently shown that NF-κB signaling is enhanced in a severe SMA mouse model^43^. Therefore, we asked if NF-κB signaling is also activated in L4–L6 DRG neurons of the mild mouse model. NF-κB is formed as homo- or heterodimers of five Rel family proteins. The most frequent subunit partners are p65 and p50, both expressed in murine lumbar DRGs; p65 is mainly expressed in small- and medium-sized neurons, while p50 expressed in all neurons^44^. We first examined mRNA levels of *Nfkb1* that encodes p105, the p50 precursor protein, and *Rela* that encodes p65. DRG mRNA samples were isolated from 3- and 9-week-old mice, and analyzed with qRT-PCR. A pronounced increase in expression of both *Nfkb1* and *Rela* was observed in SMA mice of either age group (Fig. 4a). Western blotting was further employed to examine protein level changes in DRGs with antibodies that recognize total p50 or total p65 and antibodies that recognize Ser337 phosphorylated p50 (p-p50) or Ser536 phosphorylated p65 (p-p65). To our surprise, aside from increase in the amount of total p50 or total p65, which is expected in light of the known mRNA level changes, a more robust increase in p-p50 and p-p65 levels was also detected (Fig. 4b,c). Phosphorylation is a key step in activating p50 and p65, resulting in translocation of p50 and p65 into the nucleus that initiates transcription of downstream genes. Therefore, we next asked if nuclear staining of p50 and p65 is increased in DRGs of SMA mice. DRGs obtained from 9-week-old mice were analyzed with immunofluorescence using specific mouse monoclonal antibodies against p-p50 and p-p65, respectively. Indeed, both total signal and nuclear staining were enhanced in SMA mice (Fig. 4d,e). Our data indicates that NF-κB signaling in DRGs of SMA mice is enhanced via increase in both expression and phosphorylation of the two NF-κB subunits.

### Blocking NF-κB signaling relieves pain sensitivity in SMA mice

To address whether enhanced NF-κB signaling in DRGs of the SMA mouse model is responsible for increased expression of sodium channels, we employed pyrrolidine dithiocarbamate (PDTC), a selective NF-κB inhibitor, to treat 9-week old mice. PDTC was intrathecally injected, as previously described^45^, into cerebrospinal fluid (CSF) of 9-week old SMA mice for seven consecutive days at three doses (0.01, 0.1, or 1 μg/day), and the vehicle (saline) was used as a control. As mechanical sensitivity is robustly enhanced in SMA mice and relevant to patients^25^, hereinafter, the mouse model was assessed with only von Frey filaments.

**Figure 5:**
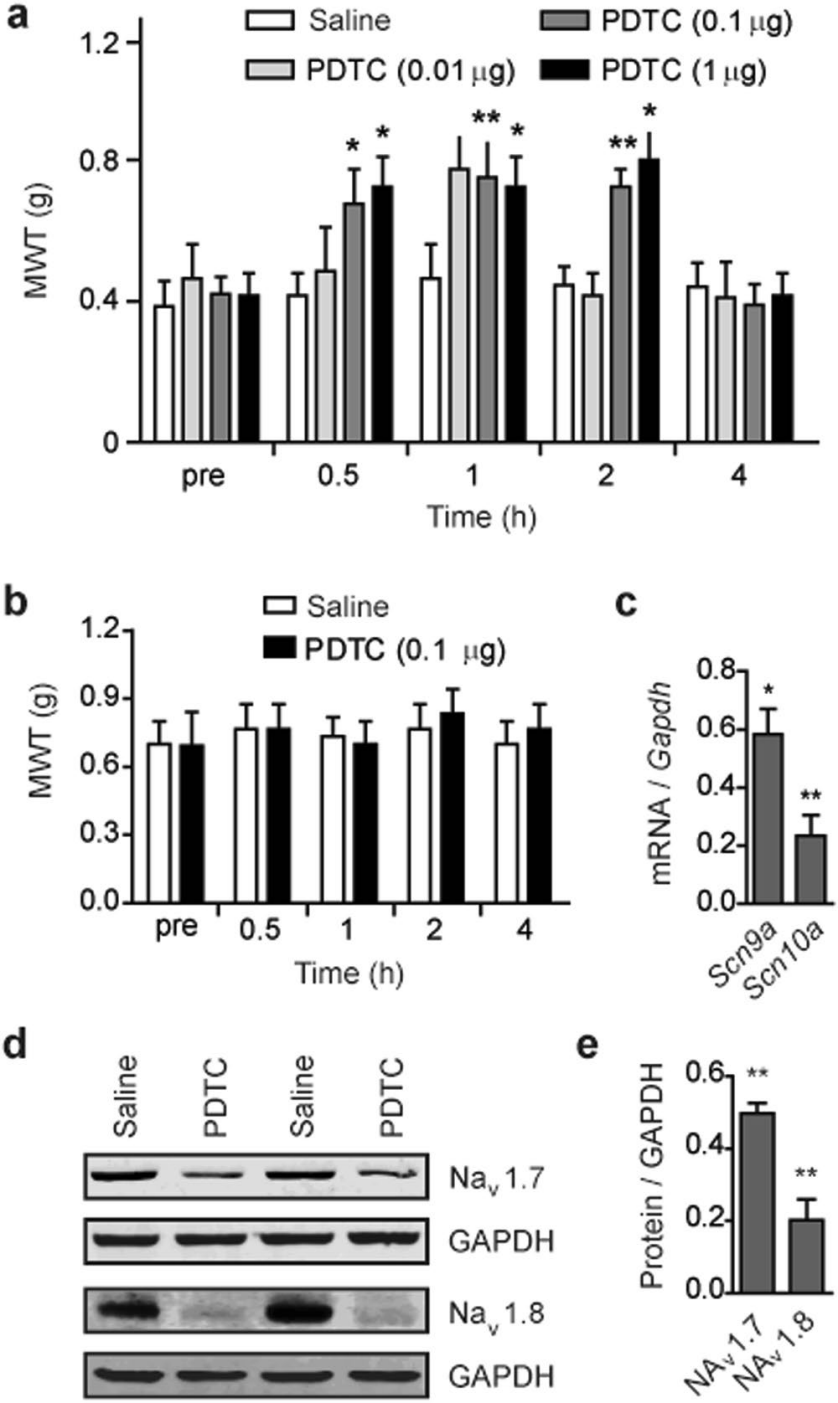
Effects of PDTC on mechanical sensitivity and expression of sodium channels in SMA mouse DRGs. (**a**) Intrathecal administration of selective NF-κB antagonist PDTC for seven consecutive days significantly increased paw mechanical withdraw threshold (MWT). Three doses (0.01, 0.1 and 1 μg/day) were analyzed with saline as a control. Von Frey test was performed at four time points: 0.5, 1, 2, and 4 hrs post last injection; n = 8 per group, *P < 0.05 and **P < 0.01 versus saline control. (**b**) NF-κB antagonist PDTC did not produce any effect in heterozygous mice (Het, n = 8). (**c**) Analysis by qRT-PCR revealed a marked decrease in both *Scn9a* and *Scn10a* mRNA levels in SMA mouse L4–L6 DRGs after treatment with 0.1 μg/day PDTC as in panel (a) (n = 3). (**d**) Western blots showed corresponding reduction in both Na_v_1.7 and Na_v_1.8 protein levels in L4–L6 DRGs of SMA mice treated with PDTC as in panel (c). (**e**) Histograms show quantitation of protein levels in panel (d); GAPDH was used as a loading control. For panels (c,e), fold changes being presented, n = 3 per group, *P < 0.05, **P < 0.01 versus Het.

Half an hour after treatment with the mid or high dose of PDTC, paw withdrawal threshold in SMA mice was significantly higher than that in saline-treated mice (Fig. 5a), whereas no effect of PDTC was observed in healthy heterozygous mice (Fig. 5b), indicating an analgesic effect of the NF-κB inhibitor. We further examined expression of *Scn9a* and *Scn10a* at mRNA and protein levels in L4–L6 DRGs by qRT-PCR and western blotting. After intrathecal treatment with 0.1 μg/day PDTC for seven consecutive days, mRNA levels of *Scn9a* and *Scn10a* in DRGs were reduced by 42% and 77%, respectively (Fig. 5c). In agreement with the mRNA result, protein levels of Na_v_1.7 and Na_v_1.8 were reduced by 51% and 80%, respectively (Fig. 5d,e). Thus, PDTC treatment reversed pain sensitivity and downregulated *Scn9a* and *Scn10a*, demonstrating that NF-κB contributes to pain hypersensitivity through regulating expression of *Scn9a* and *Scn10a* in DRGs of SMA mice.

### Adrenergic signaling is involved in pain hypersensitivity in SMA mice

**Figure 6:**
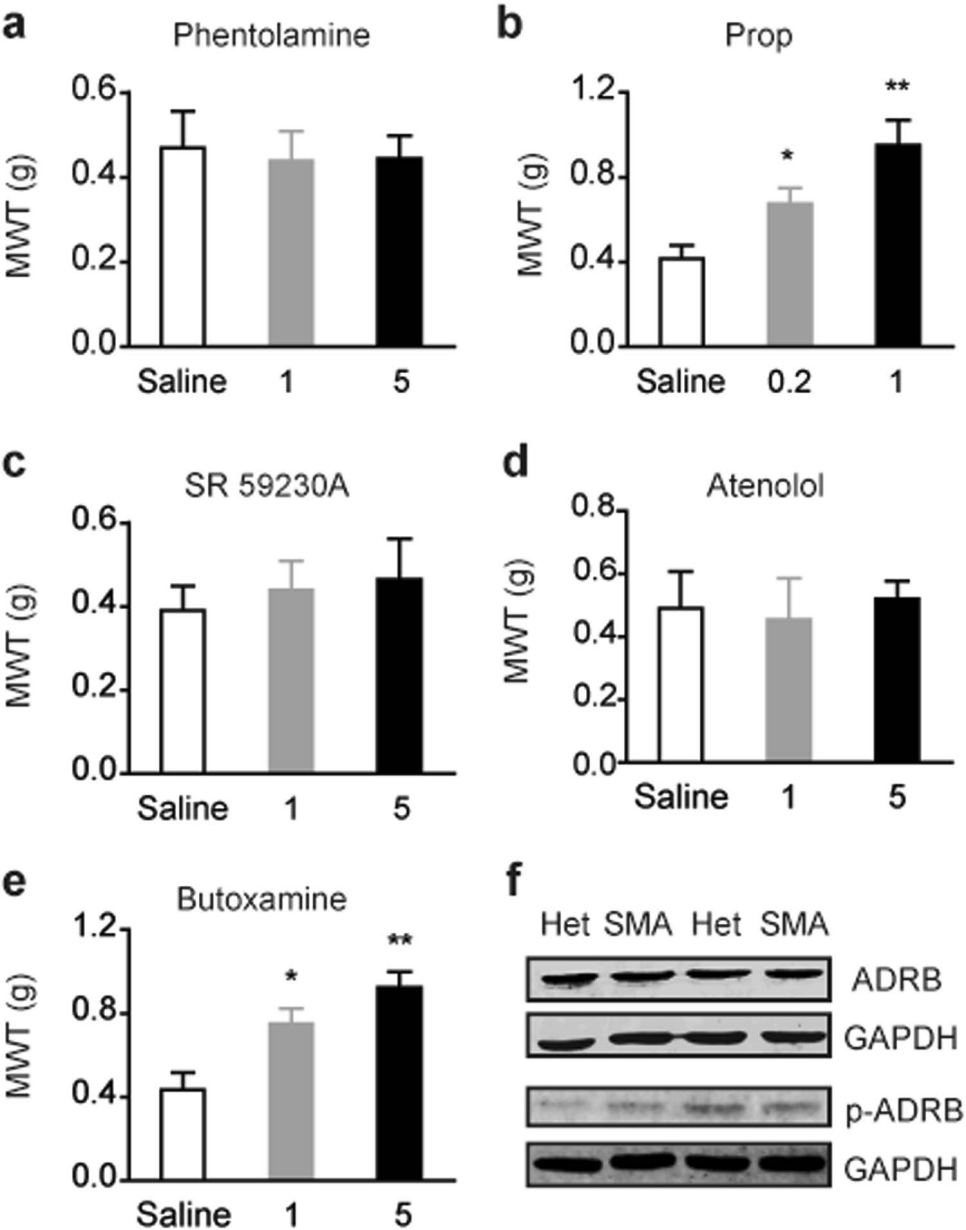
The β_2_ adrenergic receptor regulates mechanical allodynia in SMA mice. (**a**–**e**) SMA and heterozygous (Het) mice were treated with an intraperitoneal injection of nonselective α-adrenergic antagonist phentolamine, nonselective β-adrenergic antagonist propranolol (prop), or one of the three selective β_1_- (antenolol), β_2_- (butoxamine) and β_3_-adrenergic (SR 59230 A) antagonists at indicated doses (mg/kg). Thirty min after injection, mice were subjected to von Frey test and mechanical withdrawal threshold (MWT) was recorded for each mouse. For all groups, n = 8, *P < 0.05 and **P < 0.01 versus saline control. (**f**) Western blotting analysis of ADRB2 and p-ADRB2 in L4–L6 DRGs of 9-week old SMA and Het mice; no difference was observed.

Adrenergic signaling has been implicated in increased pain sensitivity through binding to either α or β receptors in human patients and animal models^46–48^. We wondered if adrenergic signaling is also involved in pain hypersensitivity in the context of SMA. To this purpose, 9-week old mice were treated with phentolamine, an antagonist of α-adrenergic receptors, at 1 and 5 mg/kg, respectively, or with propranol, an antagonist of β-adrenergic receptors, at 0.2 and 1 mg/kg, respectively. All drugs were administered intraperitoneally as described in a previous study^49^. Thirty min after treatment, mice were subjected to von Frey filament test. As shown in Fig. 6a,b, propranolol treatment increased paw withdraw threshold in SMA mice in a dose-dependent manner, and the high dose restored paw withdraw threshold to a level similar to that of heterozygous mice (Fig. 1a). On the contrary, phentolamine treatment had no effect on mechanical sensitivity. This data suggests that the hypersensitivity of nociceptive neurons observed in SMA mice involves enhanced β-adrenergic signaling. To further identify which subtype of β-adrenergic receptors accounts for mechanical allodynia and hyperalgesia observed in SMA mice, we tested atenolol, butoxamine and SR 59230 A, which are selective antagonists of the β1-, β2- and β3-adrenergic receptors, respectively. All drugs were administered via intraperitoneal injection at two doses: 1 and 5 mg/kg. Similar to propranolol, butoxamine increased paw withdrawal threshold in SMA mice in a dose-dependent manner, whereas atenolol and SR 59230 A had no effects (Fig. 6c–e), indicating that the β_2_ adrenergic receptor (ADRB2) mediates adrenergic signaling involved in SMA-associated pain hypersensitivity.

### Plasma NE levels are increased in SMA mice

**Figure 7:**
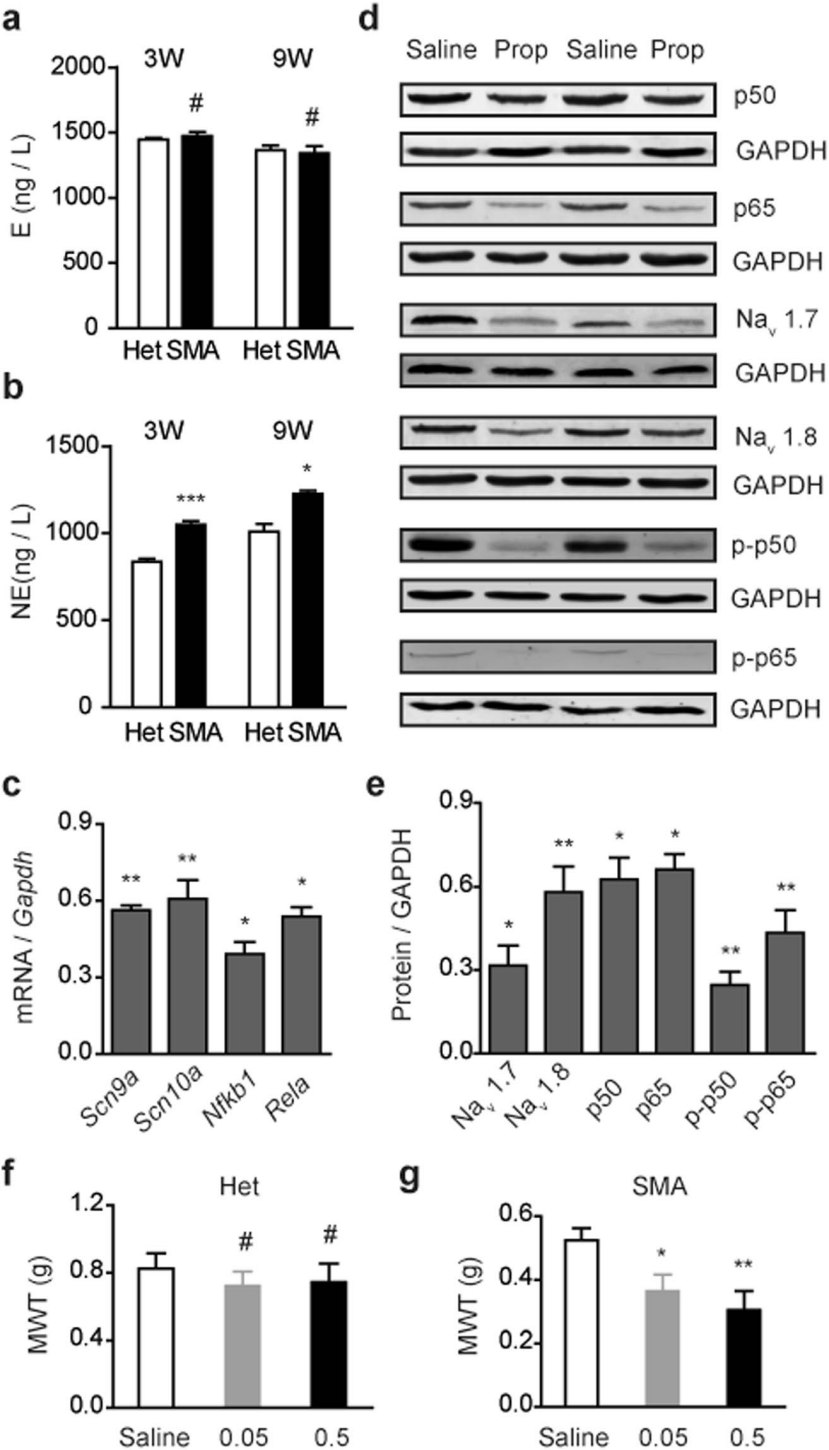
Elevated plasma NE levels in SMA mice enhance expression and phosphorylation of NF-κB subunits in DRGs. (**a**,**b**) Plasma E (n = 4) and NE (n = 10) concentrations in SMA and Het mice aged 3 or 9 weeks were measured with Elisa kits. (**c**,**d**) Mice at 9 weeks old were intraperitoneally injected with 1 mg/kg/day prop for seven consecutive days and then L4–L6 DRG RNA and protein samples were isolated for qRT-PCR analysis of expression of *Scn9a*, *Scn10a*, *Nfkb1* and *Rela* with specific primers and normalization to *Gapdh* (n = 3), or for western blotting analysis with specific antibodies against p50, p65, p-p50, p-p65, Na_v_1.7 and Na_v_1.8 (n = 3–4), respectively; GAPDH was used as a loading control. (**e)** Quantitation of data in panel (d), presented as fold changes. (**f**–**g**) NE was intraperitoneally injected into 9 weeks old mice at indicated doses (ng/g), and 30 min post injection mechanical sensitivity was assessed with von Frey test. Mechanical withdraw threshold (MWT) was recorded for each mouse. For all panels, *P < 0.05, **P < 0.01, ***P < 0.01 and ^#^*P* > 0.05 versus Het or saline treatment.

Enhanced adrenergic signaling could be caused by enhanced expression of ADRB2 itself or increased plasma levels of catecholamines. Western blot analysis using L4–L6 DRG protein samples derived from 9-week old SMA and heterozygous mice revealed no alterations of ADRB2 and phospho-ADRB2 expression (Fig. 6e). This data prompted us to examine if plasma NE and/or epinephrine (E) levels are increased in SMA mice. Using specific pAbs against NE and E, respectively, we uncovered a marked increase in the average plasma NE concentration in SMA mice assayed at the age of 3 weeks (1047 ng/L versus 835 ng/L in heterozygous mice, *P* < 0.001) and of 9 weeks (1221 ng/L versus 906 ng/L in heterozygous mice, *P* < 0.05), but no difference in the concentration of plasma E was detected (Fig. 7a,b).

NE exerts complex effects on NF-κB signaling, which vary depending on cell types^50^. In rat primary cortical neurons, NE has been observed to induce expression of IκBα, an inhibitor of NF-κB activity^51^. We hypothesized that elevation of plasma NE levels may contribute to upregulation of NF-κB in SMA mouse DRGs. Mice were treated with propranolol via intraperitoneal administration for seven consecutive days at 1 mg/kg/day, and qRT-PCR and Western blotting were performed to examine expression of p65, p50, and downstream sodium channels in L4–L6 DRGs. After treatment, both mRNA and protein levels of Na_v_1.7, Na_v_1.8, p50 and p65 in SMA mouse DRGs were markedly decreased, and the protein levels reduced by 69%, 42%, 38%, and 34%, respectively (Fig. 7c–e). More strikingly, phosphorylated p65 and p50 levels were reduced by 76% and 57%, respectively. These data demonstrate that both nuclear translocation of NF-κB subunits and their transcription activities mediate effects of NE-ADRB2 signaling on enhancement of Na_v_1.7 and Na_v_1.8 expression, resulting in hyperexcitability of nociceptive neurons in DRGs of SMA mice. Finally, we tested whether a further increase in the plasma NE concentration in SMA mice affects pain hypersensitivity. Mice at 9-week old were injected intraperitoneally with NE at two different doses (0.05 and 0.5 ng/g) and their mechanical sensitivity was assessed. Interestingly, half an hour post treatment of either dose, mechanical withdraw threshold of hind limbs of treated SMA mice was already markedly decreased (Fig. 7f,g), suggesting that NE-induced pain hypersensitivity involves both gene expression-dependent (e.g., through NF-κB signaling and expression of VGSCs) and -independent pathways.

## Discussion

**Figure 8:**
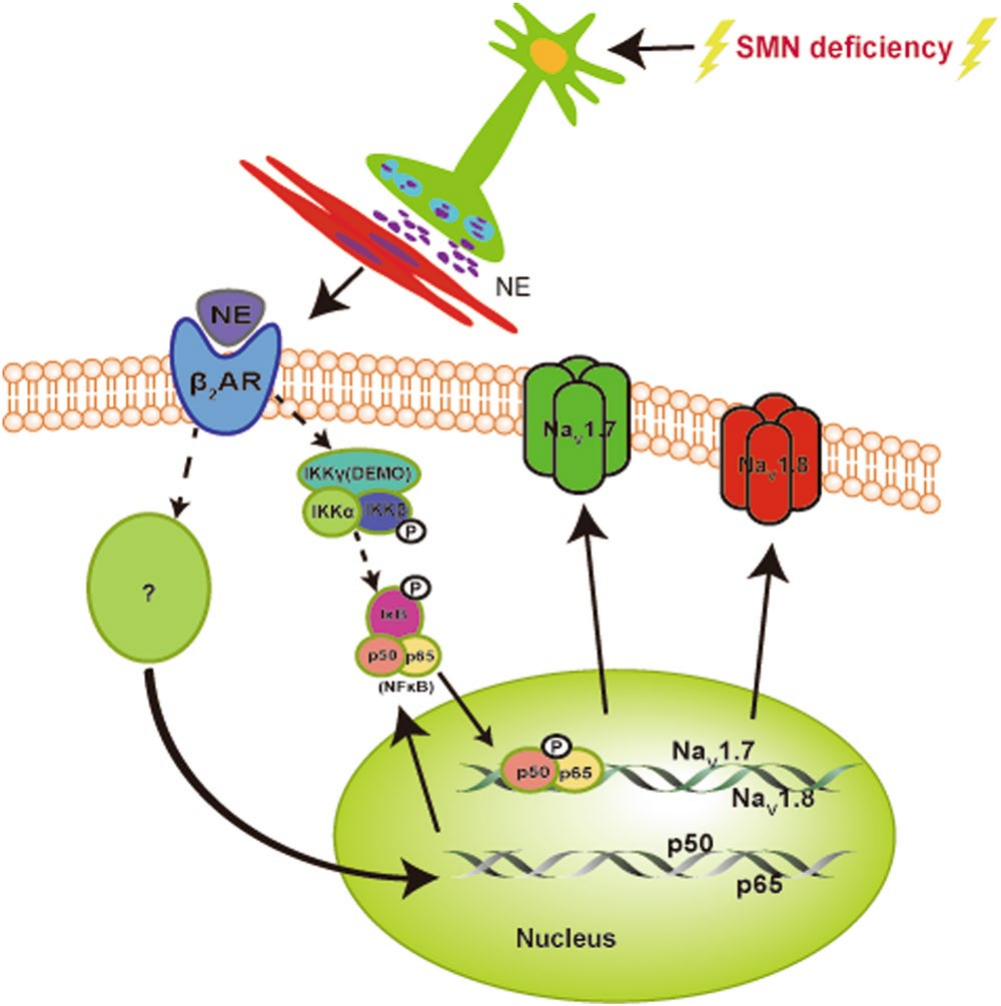
A model depicting a molecular mechanism underlying pain hypersensitivity in mild SMA mice. SMN deficiency leads to autonomic dysfunction that causes increased release of NE, which in turn, mediated by ADRB/β2AR, enhances NF-κB signaling in nociceptive DRG neurons through increasing expression and phosphorylation of p50 and p65. Enhanced NF-κB activity promotes expression of Na_v_1.7 and Na_v_1.8, contributing to hyperexcitability of these DRG neurons and pain hypersensitivity in SMA mice.

In light of a recent survey, chronic pain is a frequent issue in SMA children^25^. The causes of pain are multifactorial and vary by medical conditions. To improve pain management in SMA patients, it is necessary to elucidate signaling pathways and molecules that induce chronic pain in the disease. In this study, we revealed that the mild Taiwanese SMA mouse model displays a pronounced increase in response to both noxious and innocuous stimuli including prominent sensitization to mechanical force. Using whole-cell patch clamp recording, we found that primary nociceptive neurons in L4–L6 DRGs are hyperexcitable, which involves enhanced Na^+^ currents due to increased expression of sodium channels, particularly of TTX-sensitive Na_v_1.7 and TTX-resistant Na_v_1.8. Furthermore, we uncovered that transcription factor NF-κB is both activated and upregulated in DRGs of SMA mice. Treatment with a selective NF-κB inhibitor PDTC in SMA mice confirmed the two VGSCs are downstream targets of NF-κB signaling. Our data is consistent with earlier studies that show NF-κB as a positive regulator of Na_v_1.7 and Na_v_1.8 in rats with diabetic neuropathy or motor fiber injury^41,42^. In addition, screening with adrenergic blockers, we identified that enhanced adrenergic signaling, mediated by the β2 receptor, accounts for the increase in both phosphorylation and expression of p50 and p65 in primary nociceptive neurons of SMA mice. Finally, we unveiled that blood levels of NE, an important stress hormone and neurotransmitter as well as a pain inducer are significantly elevated in SMA mice. Therefore, we not only uncovered pain hypersensitivity as a pathological feature in a SMA mouse model, but more importantly, identified a detailed peripheral signaling cascade that induces pain hypersensitivity (Fig. 8).

We previously employed the same mild Taiwanese model in a preclinical study to first demonstrate the *in vivo* efficacy of nusinersen, the only approved drug to treat SMA, in which, an efficient correction of *SMN2* splicing and rescue of tissue necrosis, a prominent phenotypic feature of this model, were achieved^27,52^. However, to the best of our knowledge, no pathological changes in the nervous system and motor defects in the model have been previously documented; rotarod test revealed no differences in fall latency between SMA and heterozygous mice (Supplemental Fig. S3). Here we demonstrate that nociceptive DRG neurons in the model are hyperexcitable. Previously, hypoexcitability of cholinergic sensory neurons has been observed in Drosophila smn mutants^16,17^. Though the underlying mechanisms are unknown, the discrepancy should be mainly due to differences in motor circuit features between the two species. For instance, sensory and motor neurons in Drosophila are cholinergic and glutamatergic, respectively, which are opposite as in humans and mice. Nonetheless, hyperexcitability of spinal cord motor neurons as well as activation of microglia and astrocytes in severe SMA mouse models has been well documented^21,23,53^. A more recent study demonstrate that motor neurons derived from patients’ iPSCs are hyperexcitable^54^. Interestingly, the profiles of motor neuron excitability in all the previous studies are similar to what we observed in nociceptive DRG neurons. Moreover, for both neuronal cell types, hyperexcitability involves robust increase in VGSC currents. These findings suggest that hyperexcitability may be a general feature in the mammalian nervous system when SMN is deficient.

NF-κB signaling is tightly coordinately with other signaling pathways and has been implicated as a cause in multiple pain models via modulating expression of multiple pain-related genes including some VGSCs. Our data show that activation of NF-κB signaling as well as upregulation of p50 and p65 also mediates the pain hypersensitivity in SMA mice. Interestingly, while our work was in progress, two studies described a reciprocal regulation between NF-κB signaling and SMN levels^19,55^. Using mouse BV2 microglia cells, Kim and Choi reported that SMN inhibits NF-κB signaling through suppression of the E3 ubiquitin ligase activity of TRAF6 via physical interaction with the protein, a positive modulator in NF-κB signaling pathway^55^. However, no activation of p65 was observed by them when SMN was depleted in the cultured cells without addition of IL-1 in the medium, suggesting that SMN has no effect on NF-κB activity under physiological conditions. On the other hand, Arumugam *et al*. reported that NF-κB signaling inhibits expression of SMN in cultured motor neurons isolated from mouse embryos^56^. The authors also observed a reduction in phosphorylated RelA in cultured spinal cord motor neurons derived from a severe SMA mouse model, which contradicts with the result reported by Kim and Choi, which may reflect difference in cell types used in the two studies. We have recently shown that a severe mouse model develops systemic inflammation with enhanced NF-κB signaling in the liver, which is likely caused by both microbial infection and sterile stimuli^43^. Currently, we do not know whether systemic inflammation also occurs in the mild SMA mouse model and whether enhanced NF-κB signaling involves cell-autonomous mechanisms. However, our data demonstrate a non-cell-autonomous mechanism for enhancing NF-κB signaling via the NE/ADRB2 pathway in SMA mice.

Receptors that mediates NE-induced pain could be different from case to case. Either α1 or β2 antagonists have been frequently shown to attenuate pain hypersensitivity in different conditions^46,57,58^. Here we show that enhanced adrenergic signaling mediated by the β2 receptor is involved in pain hypersensitivity in SMA mice; and this is attributed in part to increase in the blood NE level rather than upregulation or phosphorylation of ADRB2. Interestingly, a recent study reported that agonists of β2 adrenergic signaling alleviated pain when injected into CSF^59^, suggesting the roles of β2 signaling are location-dependent.

The effects of NE/ADRB2 on NE-κB activity are complex, and could be inhibitory or stimulatory depending on cell types^50^. For example, NE inhibits NF-κB signaling through activating IkBα in rat primary cortical neurons and astrocytes^51,60^. On the contrary, NE activates NF-κB homodimer p50/p50 through promoting its nuclear translocation in rat cultured pinealocytes^61^. In the present study, we show that NE/ADRB2 signaling promotes expression as well as phosphorylation of p65 and p50 in SMA mouse DRG neurons. It has been well established that NE is a critical factor in causing allodynia and hyperalgesia. However, peripheral NE has no effect on pain in healthy subjects but aggravates pain in abnormal conditions. Indeed, intraperitoneal administration of NE has no pain-inducing effect on heterozygous SMA mice (Fig. 7f). This suggests that NE acts as an accomplice in pain hypersensitivity. In the case of SMA, lack of SMN presumably creates a permissive state that allows NE and potentially other agents to exert their pro-nociceptive roles. Our observation that administration of propranolol and butoxamine relieved allodynia and NE exacerbated allodynia in SMA mice in 30 min is in agreement with a previous report that epinephrine-induced hyperalgesia develops rapidly^46^. Take together with our finding that β2-adrenergic signaling promotes expression of NF-κB subunits, these data suggest two peripheral mechanisms present in the NE/ADRB2-induced pain hypersensitivity in SMA mice: a fast path and a slow path. The former mechanism should involve post-translational modifications of critical proteins including NF-κB subunits as well as PKA or PKC^46^, leading to a quick conformational alteration of VGSCs. The latter mechanism should involve gene expression alterations in the signaling nodes including upregulation of NF-κB, Na_v_1.7 and Na_v_1.8, and might play a more important role in persistent pain hypersensitivity in SMA mice.

Increase of plasma levels of NE, a major stress hormone, has profound effects on various tissues. For example, in the pancreas, it promotes release of glucagon, leading to glucose rise. In line with this, Bowenman *et al*. observed that blood glucagon levels are elevated in SMA mice^62^. Though the authors attribute the observation to an unbalance in populations of α- and β-cells in the islet, a persistent high level of blood NE is probably a cause as well^62^. The sympathetic nervous system and the hypothalamic-pituitary-adrenal axis are two sources of plasma NE. However, the fact that plasma E levels are not altered suggests activation of sympathetic nerves in SMA mice as the main cause. Although disturbance in the autonomic nervous system is a long-recognized feature in both SMA patients and mouse models, its role in SMA pathogenesis has been underappreciated^11^. Taking into account the model used in our study being a very mild one, autonomic dysfunction may be a serious issue in the context of SMA.

Although we revealed a cell-non-autonomous pathway that mediates pain hypersensitivity in mild SMA mice through activating primary nociceptive neurons (Fig. 8), the mechanisms driving SMA-associated pain hypersensitivity are undoubtedly complex. For example, we found that expression of several pain-associated potassium channel genes is downregulated in the DRGs of the SMA mouse model (Supplemental Fig. S4), which may also contribute to increased excitability of the DRG neurons.

In light of our data, several key questions remain to be addressed. First, the source of increased plasma NE levels needs to clarified. If indeed an increase in release from autonomic nerves occurs, then what is the trigger? Neuropathic or inflammatory? It is also intriguing to find out whether psychological factors such as anxiety play a role. In addition, it is important to understand to what extent the observations made in the mild mouse model are directly relevant to SMA patients. Nonetheless, considering that SMA mouse models have been widely used for drug development, leading to identification of nusinersen and many others that are in clinical trials, key genes in our study may provide potential drug targets for pain relief in patients with SMA. For example, high-throughput screens or targeted approaches can be designed to identify molecules that suppress NF-κB activities and/or excitability of primary nociceptive neurons in DRGs. Particularly, the *SCN9A* gene represents an ideal target for treating chronic pain in the context of SMA as well other diseases as disruption of this gene alone leads to lack of pain but appears not to cause other health issues except anosmia^63,64^.

## Methods

### Animals

The mild Taiwanese SMA mouse model (*Smn*^−/−^, *SMN2*^2TG/2TG^) in FVB background, originally developed by Hsieh-Li *et al*., was obtained from Cold Spring Harbor Laboratory (USA)^26,27^. The model contains a deletion of exon 7 in the *Smn* gene and carries four copies of the human *SMN2* transgene. Mice were bred and handled in accordance with the guidelines and protocols approved by the Institutional Animal Care and Use Committee of Soochow University (Suzhou, China) and the International Association for the Study of Pain. Only male mice were used in this study to avoid effects caused by sex differences.

### Behavioral tests

For analysis of mechanical sensitivity in SMA mice, von Frey filament test was employed as previously described^65^. Each animal was placed in a plastic cage with metal mesh floor and a series of von Frey filaments, 0.07 to 1.4 g (Danmic Global, San Jose, CA, USA), were applied from underneath. Pricking area was the plantar surface of right hind paws (with no fur). Paw withdrawal was regarded as a positive reaction, and mechanical sensitivity is represented by withdrawal threshold, i.e. the lowest force (g) that causes at least five positive reactions in a total of ten trials. Hargreaves test was used to assess heat sensitivity. Each mouse was placed on a glass floor maintained at 25 °C in a plastic box and its right hind paw withdrawal latency to a radiant heat (55 °C) source (Hargreaves apparatus, IITC Life Science, Woodland Hill, CA, USA) was measured from three trials; a nonresponse, which rarely occurs, was recorded as a maximal 20 s. Assessment of acetone-, formalin- and capsaicin-induced nocifensive behaviors (licking, lifting and flicking) was performed as previously described with modified doses^66^. For acetone test, 25 μl acetone was applied to the plantar surface of the right hind paw. For capsaicin and formalin tests, each mouse was injected intraplantarly to the right hind paw with either 10 µl capsaicin solution at 0.2 µg/µl, or with 20 µl of 5% formalin solution.

### Dissociation of DRG neurons and whole-cell patch clamp recording

The procedures including all used solutions were the same as in a previous study^67^. In brief, mice were sacrificed with cervical dislocation, and lumbar-segment L4–L6 DRGs were dissected out and cleaned in a dissecting solution. After treatment with collagenase D/trypsin (Sigma-Aldrich, St. Louis, MO, USA) and then DNase I (Sigma-Aldrich), single cell-suspension was obtained by trituration of DRGs through a glass pipette and placed on acid-cleaned glass coverslips. For measurement of excitability, cells were superfused with an external solution. The recording pipette was filled with a pipette solution and the voltage was clamped at −60 mV. Action potential, evoked by intracellularly injected currents, was recorded with a HEKA EPC10 patch-clamp amplifier (HEKA, Holliston, MA, USA) and analyzed with FitMaster (HEKA). For isolation of Na^+^ currents, the external solution for superfusing cells and pipette solution filled in the recording pipette were slightly different^67^. Na^+^ currents were elicited by test potentials from −70 to +50 mV in 10-mV increments with a duration of 80 ms, and recorded with the same amplifier. Current density (pA/pF), i.e. the ratio of a current amplitude to the whole cell membrane capacitance of a cell, was calculated to control cell-size differences.

### RNA isolation and qRT-PCR

Total RNA was extracted from DRG samples using Trizol (Invitrogen, Carlsbad, CA, USA) and one microgram of each RNA sample was used for first-strand cDNA synthesis in a 20-μl reaction with M-MLV (H-) reverse transcriptase (Vazyme, Nanjing, Jiangsu, China). The expression levels of *Scn3a*, *Scn9a*, *Scn10a*, *Scn11a*, *Nfkb1* and *Rela* were quantitated by a real-time RT-PCR analysis using SYBR Green I detection kit (Roche, Indianoapolis, IN, USA). Data were normalized to the housekeeping gene *Gapdh*. Primers for *Scn3a* were Scn3a-F (5′-TTGCTGCTATCGAAAAGCGTG-3′) and Scn3a-R (5′-GCACTGAATCGAAAAATTGCCT-3′), for *Scn9a* were Scn9a-F (5′-CAGCAAAGAGAGACGGAACC-3′) and Scn9a-R (5′-CCCTCAGTGTCCGTAGAGATT-3′), for *Scn10a* were Scn10a-F (5′-AATCAGAGCGAGGAGAAGACG-3′) and Scn10a-R (5′-CTAGTGAGCTAA GGATCGCAGA-3′), for Scn11a were Scn11a-F (5′-CTTTGGCTGCAATAGAGAAGCG-3′) and Scn11a-R (5′-CGTCGCCATAGA GCTTAGGTA-3′), for *Nfkb1* were Nfkb1-F (5′-ACTGCCGGGATGGCTACTAT-3′) and Nfkb1-R (5′-TCTGGATTCGCTGGCTAATGG-3′), for *Rela* were Rela-F (5′-ATGGCAGACGATGATCCCTAC-3′) and Rela-R (5′-CGGAATCGAAATCCCCTC TGTT-3′), and for *Gapdh* were Gapdh-F (5′-CCGAGACAAAATGGTGAAGGT-3′) and Gapdh-R (5′-CGTGAGTGGAGTCATACTGGAA-3′).

### Western blotting

Protein samples were separated by 10% SDS-PAGE and electroblotted onto PVDF membranes (Roche). After blocking, the blots were then incubated with monoclonal or polyclonal primary antibodies (mAb or pAb), followed by secondary IRDye® 680RD goat anti-mouse (1:5000) or goat anti-rabbit (1:5000) antibody (LI-COR Biosciences, Lincoln, NE, USA). Rabbit anti- Na_v_1.7 (1:500) and anti-Na_v_1.8 (1:500) pAbs were purchased from Alomone Labs (Jerusalem, Israel); mouse anti-p50 (1:200), anti-p65 (1:200) and anti-p-p50 (1:500) mAbs were purchased from Santa Cruz Biotechnology (Santa Cruz, CA, USA); rabbit anti-p-p65 (1:500) mAb were purchased from Cell Signaling Technology (Danvers, MA, USA); mouse anti-ADRB2/β_2_AR (1:1000) mAb and rabbit anti-p-ADRB2/β_2_AR (1:500) pAb were purchased from Proteintech (Wuhan, Hubei, China) and Sigma-Aldrich (USA), respectively; mouse anti-GAPDH (1:1000) and anti-SMN (1:1000) mAbs were purchased from MultiSciences (Hangzhou, Zhejiang, China) and BD Biosciences (San Diego, CA, USA), respectively. Protein signals were analyzed with an Odyssey Infrared Imaging System (LI-COR, Lincoln, NE, USA).

### Immunofluorescence

Lumber DRGs (L4–L6), obtained from 9-week old mice, were fixed with 4% formaldehyde in phosphate-buffered saline overnight and then soaked in 30% sucrose (w/v) at 4 °C overnight. DRGs were embedded in paraffin blocks, and 2-μm sections were cut and treated with 10 mM sodium citrate (pH 6.0) for antigen retrieval. After blocked in 5% (g/v) bovine serum albumin, sections were incubated with a specific primary antibody, followed by incubation with an Alexa Fluor 488-conjugated goat anti-mouse or anti-rabbit secondary antibody, or with an Alexa Fluor 555-conjugated goat anti-mouse or anti-rabbit secondary antibody. DAPI was used for counterstaining nuclei. Primary antibodies were the same as those used in Western blotting analysis except mouse anti-p-p65 mAb (Santa Cruz Biotechnology) for detecting phosphorylated p65. Mouse anti-NeuN (Millipore, Burlington, MA, USA) and rabbit anti-NeuN (Abcam, Cambridge, MA, USA) mAbs were used to label neuronal cells. All primary antibodies were diluted to 1:50, and secondary antibodies diluted to 1:200. Confocal immunofluorescence imaging was collected at dimensions 1024 × 1024, 8-bit and zoom 20X with a pixel dwell time of 3.15 μs/pixel on an LSM700 confocal microscope (Carl Zeiss, Germany).

### Measurement of plasma NE and E

Mice were anesthetized with 2% isofluorane and blood samples were collected from orbital sinus. After spinning, plasma was immediately aliquoted and stored at −80 °C. Plasma NE and E levels were measured using Elisa kits with biotinylated anti-NE and anti-E pAbs (Nanjing Jiancheng Bioengineering Institute, Jiangsu, China), respectively.

### Drug treatments

PDTC, Phentolamine, Propranolol, atenolol, SR5923A, butoxamine and NE were purchased from Sigma-Aldrich and freshly prepared in 0.9% saline. PDTC was injected intrathecally for consecutive 7 days with one injection per day and other drugs were injected intraperitoneally; the routes of drug administration and doses used in this study are based on prior reports^45,49^.

### Statistics

Data are expressed as means ± standard errors of the mean. Statistical differences between groups were analyzed with two-tailed t test (two means) or one-way ANOVA using software SPSS 16.0 (IBM, USA); *p* < 0.05 was considered statistically significant.

## Supplementary information

Supplemental Figures
