# Supplementary material for: SMN deficiency causes pain hypersensitivity in a mild SMA mouse model through enhancing excitability of nociceptive dorsal root ganglion neurons
Source: Sci Rep. 2019 Apr 24;9:6493. doi: 10.1038/s41598-019-43053-5 (PMC6482187; doi:10.1038/s41598-019-43053-5)
Supplement: Supplementary file 1 — Supplemental Figures [file 41598_2019_43053_MOESM1_ESM.pdf]

SMN deficiency causes pain hypersensitivity in a mild SMA mouse model through enhancing excitability of nociceptive dorsal root ganglion neurons

Ruobing Qu[1,2,#], Fuping Yao[1,2,#] Xiaomin Zhang[1,2,#], Yuan Gao[1,2], Tong Liu[1,2], Yimin Hua[1,2,*]

[1] Jiangsu Key Laboratory of Neuropsychiatric Diseases, Department of Neurology and Suzhou Clinical Research Center of Neurological Disease, the Second Affiliated Hospital of Soochow University, 1055 Sanxiang Road, Suzhou 215004, China

[2] Institute of Neuroscience, Soochow University, 199 Renai Road, Suzhou, Jiangsu 215123, China

[#] These authors contributed equally to this study

*To whom correspondence should be addressed at: Institute of Neuroscience, Soochow University, 199 Renai Road, Suzhou, Jiangsu 215123, China. Tel: +86 51265881261; Fax: +86 51265883602; Email: ymhua@suda.edu.cn

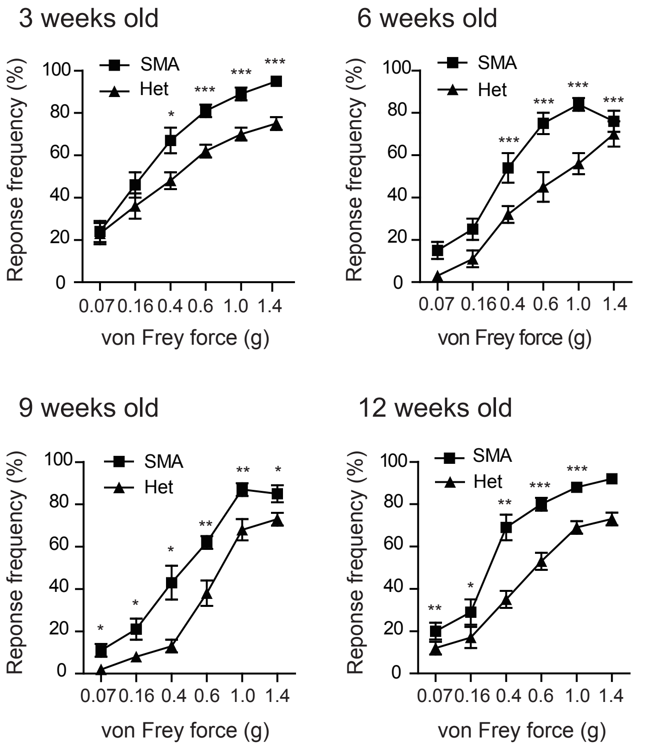

Figure S1 Mechanical allodynia occures in all age groups of SMA mice. Mechanical sensitivity of the mild Taiwanese SMA mouse model, four age groups as shown, was assessed with von Frey filaments (0.07 - 1.4 g) as in Figure 1A. For each force, each mouse was tested for 10 times. Paw withdrawal was regarded as a positive reaction, and response frequency (%) was shown on the Y-axis. Heterozygous (Het) mice were used as controls. Data are expressed as means + standard errors of the mean; n = 6 for all SMA and Het groups; *, $P < 0.05$; **, $P < 0.01$; ***, $P < 0.001$, SMA versus Het.

Qu et al._ Supplemental
Figure S2

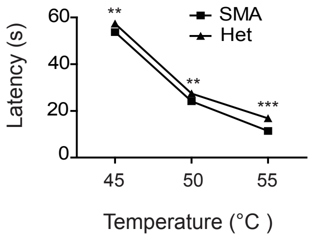

Figure S2 The mild Taiwanese SMA mouse model presents with thermal allodynia and hypergelsia. Thermal sensitivity of mild SMA mice at 9 weeks old was assessed with a conventional hot plate test at 45 ºC, 50 ºC and 55 ºC, modifid from a previous report (Yalcin I, et al., 2009, J. Pain). The withdraw latency of the right hind paw was calculated from 3 trials for each mouse to a test temperature. A maxiaml withdraw latency (20 s, 30 s or 60 s) was empirically set for a nonresponse to each test temperature (45 ºC, 50 ºC or 55 ºC), respectively. Heterozygous (Het) mice were used as controls. Data are expressed as means + standard errors of the mean; n = 6 for all SMA and Het groups; **, $P < 0.01$; ***, $P < 0.001$, SMA versus Het.

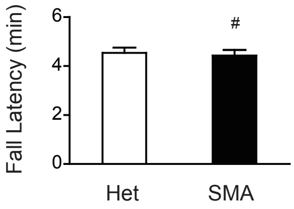

Figure S3 No differences in latency to fall were detected between mild Taiwanese SMA mice and their heterozygous (Het) controls in rotarod test. Post two-day training, each mouse at 9 weeks old was placed on the rod of a Mouse Rota-Rod 47600 instrument (Ugo Basile, Italy) at a constant rate of 25 rpm in one direction for 5 min. The average latency to fall for each mouse was calculated from three trials. Data are expressed as means + standard errors of the mean; n = 7 in each group; #, $P > 0.05$, SMA versus Het.

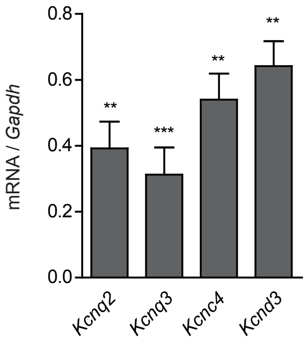

Figure S4 Four genes that encode pain-related potassium channel proteins were downregulated in DRGs of SMA mice. DRG RNA smaples of lumbar segments L4-L6 from 9-week-old SMA and heterozygous mice were isolated and analyzed by qRT-PCR. Primers for *Kcnq2* were Kcnq2-F (5' -TGACTGCCTGGTACATTGGC-3') and Kcnq2-R (5'-CTCTTGGACTTTCAGGGCAAA-3'), for *Kcnq3* were Kcnq3-F (5'-GAGCCGACAAAGACGGGAC-3') and Kcnq3-R (5'-TTGGCGTTGTTCCTCTT GACT-3'), for *Kcnc4* were Kcnc4-F (5'-TTGACCGAAACGTGACGGAG-3') and Kcnc4-R (5'-TGTA GGTAAGAATGGGCTCTGT-3'), and *for Kcnd3* were Kcnd3-F (5'-GCTCCAGCGGACAAGAACAA-3') and Kcnd3-R (5'- CTACCCAGCAAGGTGTCGG-3'). Data (n = 3) were normalized to the *Gapdh* gene. $^{**}P < 0.01$, $^{***}P < 0.001$ SMA versus Het.
